# Machine Learning–Based Motor Reserve Index and Nigral Free Water in Older Adults

**DOI:** 10.1093/geroni/igaf122.2883

**Published:** 2025-12-31

**Authors:** Edward Ofori, Alexander Belnavis, Roxana Burciu, Sydney Schaefer

**Affiliations:** Arizona State University, Phoenix, Arizona, United States; Arizona State University, Phoenix, Arizona, United States; University of Delaware, Newark, Delaware, United States; Arizona State University, Tempe, Arizona, United States

## Abstract

Objective To develop and test a machine learning–based, Motor Reserve Index (MRIx), integrating a comorbidity-based frailty measure, gait parameters, balance measures, and sex, and to evaluate its associations with free water in the anterior/posterior substantia nigra and amyloid burden. Methods De-identified data from the Health and Aging Brain Study: Health Disparities (HABS-HD) were collected from 84 older adults (mean age = 60.6 ± 9.5 years; ∼60% female). Frailty was determined via sum of chronic conditions (e.g., hypertension, cancer, diabetes, stroke, and kidney disease). Lower extremity motor measures included mean gait speed and inter-trial variability, timed up and go trial variability, Short Physical Performance Battery performance and PET SUVr (florbetaben uptake; PET-FBB) were also computed. An unsupervised dimensionality-reduction method yielded the MRIx from these features. Pearson correlations were used to test MRIx against nigral free-water measures, followed by correlations of anterior and posterior nigral free-water with PET FBB. Significance was set at p < 0.05. Results Participants with lower MRIx (suggesting diminished motor reserve) exhibited higher posterior nigral values (r = −0.29, p < 0.05). Additionally, posterior nigra free-water was positively related to Global FBB SUVR (r≈0.26, p≈0.02), indicating that elevated posterior nigral free water corresponded with greater amyloid signal. Anterior nigra showed no significant associations with MRIx or PET FBB SUVRs. Conclusion These preliminary data indicate that a machine learning–derived Motor Reserve Index, reflecting multiple factors including frailty and gait, may offer insights into posterior nigral changes and amyloid pathology in older adults.

